# Negative Risk Markers for Cardiovascular Risk Evaluation in Chinese Adults

**DOI:** 10.3389/fcvm.2022.800671

**Published:** 2022-03-15

**Authors:** Lizhan Bie, Jingya Niu, Shujing Wu, Ruizhi Zheng, Min Xu, Jieli Lu, Tiange Wang, Zhiyun Zhao, Shuangyuan Wang, Hong Lin, Meng Dai, Di Zhang, Yuhong Chen, Yufang Bi, Weiqing Wang, Guang Ning, Mian Li, Yu Xu

**Affiliations:** ^1^Department of Endocrine and Metabolic Diseases, Shanghai Institute of Endocrine and Metabolic Diseases, Ruijin Hospital, Shanghai Jiaotong University School of Medicine, Shanghai, China; ^2^Shanghai National Clinical Research Center for Metabolic Diseases, Key Laboratory for Endocrine and Metabolic Diseases of the National Health Commission, Shanghai National Center for Translational Medicine, Ruijin Hospital, Shanghai Jiaotong University School of Medicine, Shanghai, China; ^3^Jiading District Central Hospital Affiliated Shanghai University of Medicine and Health Sciences, Shanghai, China

**Keywords:** cardiovascular disease, negative risk marker, lipoprotein(a), electrocardiogram, carotid intima-media thickness

## Abstract

**Background:**

The atherosclerotic cardiovascular disease (ASCVD) risk predicted by traditional risk factors is used to guide preventive treatment. We aimed to investigate whether preferable levels of non-traditional emerging risk factors (i.e., negative risk markers) could downgrade the predicted ASCVD risk beyond traditional risk factors.

**Methods:**

A total of 7,568 Chinese adults aged ≥ 40 years were followed up during 2010–2015. Negative risk markers including non-traditional lipids, urinary albumin-to-creatinine ratio, electrocardiogram (ECG), and measurements of atherosclerosis were evaluated using diagnostic likelihood ratio (DLR) and continuous net reclassification index (NRI) for their ability to downshift predicted CVD risk in the overall study population and in participants with intermediate (traditional risk factor predicted ASCVD risk 7.5% to 19.9%) or high risk (≥20%).

**Results:**

During a median follow-up of 4.5 years, 416 participants developed CVD events including non-fatal myocardial infarction, non-fatal stroke, and cardiovascular death. Among negative risk markers examined, lipoprotein(a) ≤ 10th percentile (5 mg/dL), normal ECG, and carotid intima-media thickness (CIMT) ≤ 25th percentile (0.5 mm) provided moderate CVD risk reclassification and downward changes in pre- to post-test risk on top of the traditional CVD risk factors, especially in high-risk participants. The DLRs were 0.41, 0.75, and 0.41, and the NRIs were 18, 22, and 14% for lipoprotein(a), ECG, and CIMT, respectively in high-risk participants.

**Conclusions:**

Lipoprotein(a) ≤ 5 mg/dL, normal ECG, and CIMT ≤ 0.5 mm might be used as negative non-traditional risk markers to correctly downgrade predicted ASCVD risk in Chinese adults.

## Introduction

Cardiovascular disease (CVD) is a major health challenge for the modern society. The total prevalent cases of CVD are ~523 million globally in 2019 and 18.6 million people die of CVD each year (1). The heavy burden of CVD is attributable to the increasing prevalence of hypertension, diabetes, dyslipidemia, and chronic kidney disease, et al. (1). Risk assessment tools for CVD development are important in predicting future CVD events for early intervention (2, 3). A well-known example is the Pooled Cohort Equations (PCEs) for 10-year atherosclerotic CVD (ASCVD) risk calculated using traditional CVD risk factors recommended by the American College of Cardiology/American Heart Association (ACC/AHA) (2). Preventive medications such as statins are recommended in people with elevated ASCVD risk predicted using the PCEs (4, 5). However, the absence of non-traditional CVD risk factors often predicted a low risk of CVD, regardless of PCE score (6). A previous study found that a coronary artery calcium (CAC) score of 0 was associated with a reduced CVD risk and thus defined CAC = 0 as a negative risk marker (7). For individuals at intermediate ASCVD risk estimated by the PCE score (7.5–19.9%) and without coronary artery calcium, statin therapy may be withheld or delayed (8). Therefore, negative risk markers can be used to reduce overtreatment.

China has the largest number of people suffering from CVD worldwide (1). Because the PCEs were developed in US cohorts, it may overestimate ASCVD risk in Chinese adults (9). Chinese adults might receive preventive treatment based on an overestimated risk by PCEs. Therefore, it is demanded to identify individuals with high-predicted risk but low-observed risk in Chinese adults. Negative risk markers have shown significant potentials in downgrading the predicted 10-year ASCVD risk, raising the question whether there are other negative risk markers to help identify the true “low risk” population in addition to a CAC=0. Therefore, in the current study, we aimed to examine the ability of several potential negative risk markers to downgrade the predicted 10-year ASCVD risk in Chinese adults.

## Method

### Study Population

A total of 10,375 community residents aged 40 years or older were recruited from Jia Ding District, Shanghai, China between March and August 2010. During August 2014 and May 2015, participants were invited to take part in a follow-up examination for the development of CVD events including myocardial infarction (MI), stroke, and cardiovascular death. The study design and data collection procedures were described previously (10, 11). We excluded 306 participants with a history of CVD, 147 participants aged 80 years and older, 756 participants with missing data on negative risk markers at baseline and 1,580 participants with missing data on CVD outcomes at follow-up. Therefore, 7,586 participants were included in the current analysis.

The study protocol was approved by the Institutional Review Board of the Ruijin Hospital affiliated to Shanghai Jiaotong University School of Medicine. Written informed consent was obtained from each participant before data collection.

### Baseline Examination

Baseline characteristics including sociodemographic variables, history of chronic diseases and medications, and lifestyle habits, et al. were evaluated using a standard questionnaire administered during a face-to-face interview. Blood pressure was measured on the non-dominant arm of each participant after at least 10 min of sitting rest using an automated device (OMRON Model HEM- 752 FUZZY; Omron Co., Dalian, China) three times consecutively with 1-min intervals, and the mean of the three measurements was used for analysis.

Venous blood samples were drawn in the morning from each participant after at least a 10-h overnight fast. An oral glucose tolerance test was conducted in participants without diabetes history and 2-h post-load blood samples were collected. Fasting and post-load blood glucose levels were measured on an autoanalyzer (Modular P800; Roche, Basel, Switzerland) using the glucose oxidation method. Diabetes was defined as a fasting blood glucose level ≥126 mg/dL and/or a post-load glucose level ≥200 mg/dL and/or taking any glucose-lowering medications. Concentrations of fasting serum total cholesterol, high-density lipoprotein cholesterol (HDL-c), lipoprotein(a), apoB, apoA-I were measured on an autoanalyzer (Modular E170; Roche) using the chemiluminescence method. More details on lipid measurements are described previously (12).

The first morning spot urine samples were obtained from each participant. The urinary albumin levels were measured by the immunoturbidimetric method (Beijing Atom High-Tech, Beijing, China) and urinary creatinine levels were measured by the Jaffe's kinetic method on an automatic analyzer (Hitachi 7600-020, Tokyo, Japan). Urinary albumin-to-creatinine ratio (UACR) (mg/g) was calculated as the urinary albumin concentration divided by the urinary creatinine concentration.

Electrocardiograms (ECG) were recorded according to a standard protocol with participants in the supine position using a 12-lead ECG machine (CAM14, GE, US). Computer-assigned Minnesota Code (MC) to electrocardiographs of each participant was used for categorization. Carotid intima-media thickness (CIMT) measurements were carried out by an experienced sonographer using a high-resolution B-mode tomographic ultrasound system (Esaote Biomedica SpA, Italy) with a linear 7.5-MHz transducer. The CIMT was measured on the far wall of the right and left common carotid arteries, 1.5 cm proximal to the bifurcation. The distance from the leading edge of the first echogenic line to that of the second echogenic line at the end of diastole was calculated as the CIMT of either side. The brachial-ankle pulse wave velocity (baPWV) and the ankle-brachial index (ABI) were measured using a Colin VP-1000 device (Model BP203RPE II, form PWV/ABI, China) after 10 min of rest. After placing the cuffs on the right and left upper arms and the right and left ankles, pulse waves were obtained simultaneously. The time delay in obtaining pulse waves and the distance of the right and left upper arms to the right and left ankles were included in the calculation of the right and left baPWVs. ABI was calculated as the ratio of ankle systolic BP to arm systolic BP. The larger CIMT or baPWV at either side was used for analysis.

### Negative Risk Markers

Negative risk markers were chosen based on previous evidence of significant associations with CVD risks. Several negative risk markers were examined in the current study, including non-traditional lipids, UACR, ECG, and measurements of atherosclerosis. For risk markers that have definite clinical cutoff points such as UACR, ECG, and ABI, we used these cutoffs to define “normal” or “negative.” For example, UACR <30 mg/g is regarded as normal according to the Kidney Disease Improving Global Outcomes (KDIGO) clinical practice guideline (13). In addition, because previous studies indicate that the risk extends below this level, we also categorized UACR as “optimal” values below 10 mg/g (14). For risk markers that do not have definite clinical cutoff points such as non-traditional lipids, PWV, and CIMT, we used the lowest or highest quartile to indicate an optimal level. In addition, for lipids, we also used the lowest or highest 10 percentile to indicate a more stringent optimal level. Specifically, they were: (1) lipoprotein(a) ≤ 25th percentile (9 mg/dL) or ≤ 10th percentile (5 mg/dL), (2) apoB ≤ 25th percentile (0.81 g/L) or ≤ 10th percentile (0.69 g/L), (3) apoA-I ≥75th percentile (1.43 g/L) or ≥90th percentile (1.60 g/L), (4) UACR <30 mg/g or <10 mg/g, (5) normal ECG, (6) normal corrected QT interval (men: 390–450 ms, women: 390–460 ms), (7) CIMT ≤ 25th percentile (0.5 mm), (8) baPWV ≤ 25th percentile (1,350 cm/s), and (9) normal ABI (≥0.90 and ≤ 1.30) at both sides.

### CVD Outcomes

The development of CVD events including non-fatal MI, non-fatal stroke, and cardiovascular death was recorded by self-report from participants or collected from the national insurance system and local death registries. Photocopies of the participant's inpatient record, discharge summary, electrocardiogram, and imaging reports were obtained. Myocardial infarction was defined by changes in levels of troponin T and Creatine-Kinase-MB isoform, symptoms of myocardial ischemia, changes in ECG results, et al. Stroke was defined by a fixed neurological deficit lasting >24 h because of a presumed vascular cause. Cardiovascular death was defined as death due to cardiovascular causes. Two physicians independently reviewed medical charts and verified each CVD event with discrepancies resolved through discussion (15).

### Statistical Analysis

Baseline characteristics of the overall study population and participants who did or did not develop CVD events are described with continuous variables in means ± standard deviations (SD) or medians (25–75% quartile) and categorical variables in numbers (percentages). Differences between participants with and without incident CVD events were compared using student's *t*-test for continuous variables and chi-square test for categorical variables. Numbers (percentages) of CVD events in participants with and without each individual negative risk markers were calculated and compared using chi-square test. Cox regression models were used to assess the associations (hazard ratios [HRs] and 95% confidence interval [CI]) of negative risk markers with the development of CVD in the overall study population as well as in individuals with intermediate ASCVD risk (10-year predicted ASCVD risk of 7.5 to 19.9%) or high ASCVD risk (10-year predicted ASCVD risk ≥ 20%) calculated using the PCEs. Model 1 was adjusted for age and sex. Model 2 was further adjusted for other traditional CVD risk factors including current smoking, diabetes, total cholesterol, HDL-c, systolic blood pressure, and antihypertensive drug treatment.

The continuous net reclassification index (NRI) was used to measure the ability of each negative risk markers to improve the CVD risk classification beyond traditional risk factors. To do this, we established a basic logistic regression model that included traditional risk factors. The predicted CVD risks by the basic model with and without negative risk markers were compared. Because the negative risk markers are able to downgrade risk, the positive NRIs will be driven by improvements in specificity (indicating less medication-overuse).

Finally, we calculated the diagnostic likelihood ratio (DLR), which measures the impact of negative risk markers by comparing pre-test and post-test risks. We constructed logistic regression models including baseline predictors X (e.g., traditional CVD risk factors) to predict pre-test CVD risk, and logistic regression models including predictors X and predictor Y (e.g., individual negative risk markers) to predict post-test CVD risk. The multivariable-adjusted DLR is then calculated by subtracting the pre-test risk model from the post-test risk model. More details about the DLR calculation have been reported in the previous literature (7). A DLR > 1 indicates that post-test risk is higher than pre-test risk (i.e., test risk markers have no effect on risk reduction), whereas a DLR <1 indicates that post-test risk is lower than pre-test risk (i.e., test risk markers may have effect on risk reduction).

All statistical analyses were performed using R 4.0.2 (http://www.r-project.org/) and SAS version 9.4 (SAS Institute Inc., Cary, North Carolina). All analyses were two-sided and a *p-value* of < 0.05 was considered statistically significant.

## Results

### General Characteristics

A total of 416 participants developed CVD events during a median of 4.5 years of follow-up. Table 1 shows the baseline characteristics of the study participants. Overall, the average age was 57.9 ± 9.0 years and 37.8% were men. Approximately 23.7% of the participants were current smokers, 17.7% had diabetes, and the median (interquartile range) estimated 10-year ASCVD risk was 6.7% (2.7 to 15.1%). Generally, levels of systolic blood pressure, lipoprotein(a), baPWV, CIMT, QTc interval, and UACR, and proportions of participants with diabetes, or abnormal ECG increased significantly in participants who developed CVD events compared with those who did not (all *p-value*s < 0.05).

**Table 1:** Characteristics of the study population at baseline.

**Characteristics**	**Total**	**Incident CVD events**		
	**(*n =* 7,586)**	**Without (*n =* 7,170)**	**With (*n =* 416)**	***p-value***
Age, years	57.9 ± 9.0	57.6 ± 8.9	63.0 ± 9.2	<0.001
Men, *n* (%)	2,867 (37.8)	2,697 (37.6)	170 (40.9)	0.202
Current smoker, *n* (%)	1,800 (23.7)	1,695 (2,367)	105 (25.2)	0.492
Type 2 diabetes, *n* (%)	1,345 (17.7)	1,218 (17.0)	127 (30.5)	<0.001
Systolic blood pressure, mmHg	141.0 ± 19.9	140.5 ± 19.7	148.9 ± 21.3	<0.001
Total cholesterol, mg/dL	206.4 ± 39.0	206.2 ± 39.0	209.9 ± 39.1	0.062
HDL cholesterol, mg/dL	51.4 ± 12.4	51.4 ± 12.5	51.6 ± 11.8	0.866
Use of antihypertensive drugs, *n* (%)	2,059 (27.1)	1,888(26.3)	171(41.1)	<0.001
10-year ASCVD risk *	6.7 (2.7, 15.1)	6.5 (2.6, 14.4)	14.5 (5.1, 27.2)	<0.001
Lp(a), mg/dL	20.9 ± 15.5	20.8 ± 15.5	22.6 ± 15.5	0.024
apoB, mg/dL	0.97 ± 0.24	0.97 ± 0.24	0.99 ± 0.24	0.087
apoA-I, mg/dL	1.26 ± 0.28	1.25 ± 0.28	1.26 ± 0.27	0.549
UACR, mg/g	4.87 (2.77, 9.06)	4.81 (2.77, 8.89)	6.00 (2.89, 13.14)	<0.001
Normal ECG, *n* (%)	3,063 (40.4)	2,930 (40.9)	133 (32.0)	<0.001
QTc interval, ms	433.2 ± 32.5	432.8 ± 32.4	439.4 ± 34.0	<0.001
baPWV, cm/s	1,599.4 ± 356.7	1,589.6 ± 349.6	1,766.8 ± 429.4	<0.001
CIMT, mm	0.58 ± 0.11	0.58 ± 0.11	0.63 ± 0.11	<0.001
Normal ABI, *n* (%)	6,898 (90.9)	6,520 (90.9)	378 (90.9)	1.000

*Data are means ± standard deviations, numbers (percentages), or medians (interquartile ranges)*.

*Estimated using the PCEs.

*CVD, cardiovascular diseases; HDL, high-density lipoprotein; Lp (a), lipoprotein(a); apoB, apolipoprotein B; apoA-I, apolipoprotein A-I; UACR, urinary albumin-to-creatinine ratio; baPWV, brachial-ankle pulse wave velocity; CIMT, carotid intima-media thickness; ABI, ankle-brachial index; ECG, electrocardiogram; QTc interval, corrected QT interval; ASCVD, atherosclerotic cardiovascular disease; PCEs, Pooled Cohort Equations*.

### Negative Risk Markers in Association With CVD Risk

Most participants had normal (93.55%) or low UACR (77.51%), or normal ABI (90.93%). Less than half participants had other negative risk markers (Table 2). The development of incident CVD events was the lowest in participants with CIMT ≤ 25th percentile (3.14%), followed by that in participants with baPWV ≤ 25th percentile (3.72%). The development of incident CVD events were significantly different in participants with *vs*. without lipoprotein(a) ≤ 10th percentile, UACR <30 mg/g or <10 mg/g, normal ECG, normal corrected QT interval, or CIMT ≤ 25th percentile.

**Table 2:** The development of CVD events in participants with and without negative risk markers.

**Negative risk markers**	**Number of participants (%)**	**Incident CVD events**, ***n*** **(%)**		
		**In participants with negative risk markers**	**In participants without negative risk markers**	***p*−*value*^a^**
**Non-traditional lipids**				
Lp(a) ≤ 25th percentile (9 mg/dL)	2,074 (27.34)	98 (4.73)	318 (5.77)	0.187
Lp(a) ≤ 10th percentile (5 mg/dL)	923 (12.17)	35 (3.80)	381 (5.72)	0.060
apoB ≤ 25th percentile (0.81 g/L)	1,991 (26.25)	96 (4.82)	320 (5.72)	0.340
apoB ≤ 10th percentile (0.69 g/L)	819 (10.80)	43 (5.25)	373 (5.51)	0.933
apoA-I ≥ 75th percentile (1.43 g/L)	1,915 (25.24)	110 (5.74)	306 (5.40)	0.760
apoA-I ≥ 90th percentile (1.60 g/L)	784 (10.33)	47 (5.99)	369 (5.42)	0.886
**Urinary ACR**				
<30 mg/g	7,097 (93.55)	364 (5.14)	52 (10.63)	<0.001
<10 mg/g	5,880 (77.51)	283 (4.81)	133 (7.80)	<0.001
**Electrocardiogram**				
Normal ECG	3,063 (40.38)	133 (4.34)	283 (6.26)	0.018
Normal QTc (men: 390–450 ms, women: 390–460 ms)	6,087 (80.23)	293 (4.81)	123 (7.41)	<0.001
**Measurements of atherosclerosis**				
baPWV ≤ 25th percentile (1,350 cm/s)	1,909 (25.16)	71 (3.72)	345 (6.08)	0.770
CIMT ≤ 25th percentile (0.5 mm)	2,991 (39.43)	94 (3.14)	322 (7.01)	<0.002
Normal ABI (0.9-1.3)	6,898 (90.93)	378 (5.48)	38 (5.52)	0.752

*Data are numbers (percentages) unless otherwise indicated*.

*^a^P-values were adjusted for age and sex*.

*CVD, cardiovascular diseases; HDL, high-density lipoprotein; Lp (a), lipoprotein(a); apoB, apolipoprotein B; apoA-I, apolipoprotein A-I; UACR, urinary albumin-to-creatinine ratio; baPWV, brachial-ankle pulse wave velocity; CIMT, carotid intima-media thickness; ABI, ankle-brachial index; ECG, electrocardiogram; QTc interval, corrected QT interval; ASCVD, atherosclerotic cardiovascular disease; PCEs, Pooled Cohort Equations*.

Table 3 shows the associations of negative risk markers with CVD risks. In the overall study population, lipoprotein(a) ≤ 10th percentile, UACR <30 mg/g or <10 mg/g, normal QTc, and CIMT ≤ 25th percentile were all associated with a significantly reduced risk of CVD events after adjustment for traditional CVD risk factors. They were also associated with a significantly reduced CVD risk in participants with high risk (10-year predicted ASCVD risk ≥20%). In addition, lipoprotein(a) ≤ 25th percentile and normal ECG also demonstrated associations with a significantly reduced CVD risk in participants with high risk. None of the negative risk markers were associated with reduced CVD risks in participants with intermediate risk.

**Table 3:** The associations of negative risk markers with CVD risk.

**Negative risk markers**	**Total (*n =* 7,586)**		**Intermediate risk (*n = * 2,248)**		**High risk (*n =* 1,299)**	
	**Model 1**	**Model 2**	**Model 1**	**Model 2**	**Model 1**	**Model 2**
**Non-traditional lipids**						
Lp(a) ≤ 25th percentile (9 mg/dL)	0.87 (0.70, 1.10)	0.82 (0.65, 1.03)	0.94 (0.63, 1.42)	0.89 (0.59, 1.34)	**0.62 (0.42, 0.91)**	**0.62 (0.42, 0.92)**
Lp(a) ≤ 10th percentile (5 mg/dL)	0.73 (0.51, 1.03)	**0.67 (0.47, 0.95)**	0.89 (0.50, 1.60)	0.80 (0.44, 1.43)	**0.41 (0.21, 0.80)**	**0.41 (0.21, 0.80)**
apoB ≤ 25th percentile (0.81 g/L)	0.87 (0.70, 1.10)	0.97 (0.74, 1.28)	1.15 (0.75, 1.77)	0.89 (0.53, 1.51)	0.68 (0.45, 1.03)	0.77 (0.48, 1.25)
apoB ≤ 10th percentile (0.69 g/L)	0.97 (0.71, 1.33)	1.12 (0.79, 1.58)	1.22 (0.68, 2.19)	0.98 (0.51, 1.90)	0.78 (0.42, 1.45)	0.90 (0.47, 1.74)
apoA-I ≥ 75th percentile (1.43 g/L)	0.95 (0.76, 1.18)	0.94 (0.72, 1.23)	0.83 (0.54, 1.28)	0.76 (0.45, 1.28)	1.25 (0.89, 1.76)	1.20 (0.79, 1.81)
apoA-I ≥ 90th percentile (1.60 g/L)	1.01 (0.74, 1.37)	1.02 (0.72, 1.46)	1.04 (0.58, 1.87)	1.02 (0.52, 2.00)	1.30 (0.80, 2.10)	1.16 (0.65, 2.07)
**Urinary ACR**						
<30 mg/g	**0.54 (0.41, 0.73)**	**0.64 (0.47, 0.86)**	0.64 (0.35, 1.18)	0.71 (0.38, 1.30)	**0.52 (0.35, 0.77)**	**0.55 (0.37, 0.82)**
<10 mg/g	**0.71 (0.57, 0.87)**	**0.80 (0.65, 0.99)**	0.70 (0.47, 1.06)	0.75 (0.50, 1.13)	0.74 (0.54, 1.03)	0.78 (0.56, 1.08)
**Electrocardiogram**						
Normal ECG	**0.80 (0.65, 0.98)**	0.85 (0.69, 1.05)	0.79 (0.53, 1.17)	0.84 (0.57, 1.26)	**0.67 (0.47, 0.96)**	**0.70 (0.49, 1.01)**
Normal QTc (men: 390–450 ms, women: 390–460 ms)	**0.65 (0.53, 0.81)**	**0.71 (0.57, 0.88)**	0.69 (0.46, 1.03)	0.74 (0.50, 1.11)	**0.61 (0.45, 0.84)**	**0.63 (0.46, 0.87)**
**Measurements of atherosclerosis**						
baPWV ≤ 25th percentile (1,350 cm/s)	1.03 (0.78, 1.36)	1.44 (1.07, 1.94)	1.04 (0.61, 1.77)	1.41 (0.80, 2.48)	/*	/*
CIMT ≤ 25th percentile (0.5 mm)	**0.67 (0.52, 0.86)**	**0.71 (0.55, 0.91)**	0.85 (0.55, 1.32)	0.84 (0.54, 1.30)	**0.38 (0.19, 0.78)**	**0.39 (0.19, 0.79)**
Normal ABI (0.9–1.3)	1.07 (0.77, 1.49)	1.09 (0.78, 1.52)	1.25 (0.63, 2.47)	1.26 (0.63, 2.48)	0.90 (0.57, 1.45)	0.94 (0.59, 1.50)

*Data are hazard ratios (95% confidence intervals). The bold values indicate statistical significance*.

*Intermediate risk: 10-year ASCVD risk 7.5% to 19.9% by the PCEs; High risk: 10-year ASCVD risk ≥ 20% by the PCEs*.

*Model 1: adjusted for age and sex*.

*Model 2: further adjusted for smoking status, diabetes, total cholesterol, HDL-c, systolic blood pressure, antihypertensive drugs*.

*Data cannot be shown due to a limited number of participants with high risk and baPWV ≤ 25th percentile (n = 45).

*CVD, cardiovascular diseases; HDL, high-density lipoprotein; Lp (a), lipoprotein(a); apoB, apolipoprotein B; apoA-I, apolipoprotein A-I; UACR, urinary albumin-to-creatinine ratio; baPWV, brachial-ankle pulse wave velocity; CIMT, carotid intima-media thickness; ABI, ankle-brachial index; ECG, electrocardiogram; QTc interval, corrected QT interval; ASCVD, atherosclerotic cardiovascular disease; PCEs, Pooled Cohort Equations*.

### CVD Risk Reclassification

We evaluated CVD risk reclassifications by negative risk markers in addition to traditional ASCVD risk factors in Table 4. Moderate and significant risk reclassifications were observed in the overall study population for negative risk markers such as UACR <10 mg/g, normal ECG, normal QTc, and CIMT ≤ 25th percentile (continuous NRI from 0.18 to 0.25). In addition to these negative risk markers, lipoprotein(a) ≤ 25th percentile or ≤ 10th percentile, UACR <30 mg/g, normal ECG, normal QTc and CIMT ≤ 25th percentile also demonstrated moderate risk reclassifications in participants with high predicted ASCVD risk (continuous NRI from 0.14 to 0.24). Only urinary ACR <10 mg/g had significant CVD risk reclassifications in participants with moderate predicted ASCVD risk.

**Table 4:** Continuous NRI for CVD risk by adding each negative risk markers to a basic model with traditional CVD risk factors.

**Negative risk markers**	**Total (*n =* 7,586)**	**Intermediate risk (*n =* 2,248)**	**High risk (*n =* 1,299)**
**Non-traditional lipids**			
Lp(a) ≤ 25th percentile (9 mg/dL)	**0.08 (0.00, 0.17)**	0.04 (−0.12, 0.21)	**0.23 (0.10, 0.37)**
Lp(a) ≤ 10th percentile (5 mg/dL)	**0.09 (0.03, 0.14)**	0.06 (−0.06, 0.17)	**0.18 (0.10, 0.27)**
apoB ≤ 25th percentile (0.81 g/L)	0.02 (−0.08, 0.11)	−0.01 (−0.19, 0.17)	0.06 (−0.10, 0.21)
apoB ≤ 10th percentile (0.69 g/L)	0.07 (−0.02, 0.16)	0.02 (−0.15, 0.20)	−0.07 (−0.22, 0.08)
apoA-I ≥ 75th percentile (1.43 g/L)	0.08 (−0.02, 0.17)	0.13 (−0.05, 0.30)	0.00 (−0.16, 0.16)
apoA-I ≥ 90th percentile (1.60 g/L)	−0.06 (−0.15, 0.04)	−0.11 (−0.29, 0.07)	−0.05 (−0.21, 0.12)
**Urinary ACR**			
<30 mg/g	0.03 (−0.04, 0.10)	0.08 (−0.04, 0.21)	**0.20 (0.07, 0.33)**
<10 mg/g	**0.18 (0.09, 0.27)**	**0.18 (0.01, 0.35)**	**0.18 (0.02, 0.34)**
**Electrocardiogram**
Normal ECG	**0.18 (0.09, 0.27)**	0.15 (−0.02, 0.32)	**0.22 (0.08, 0.37)**
Normal QTc (men: 390–450 ms, women: 390–460 ms)	**0.21 (0.12, 0.30)**	0.15 (−0.01, 0.32)	**0.24 (0.08, 0.39)**
**Measurements of atherosclerosis**
baPWV ≤ 25th percentile (1,350 cm/s)	0.07 (−0.03, 0.16)	0.14 (−0.04, 0.31)	/*
CIMT ≤ 25th percentile (0.5 mm)	**0.25 (0.16, 0.34)**	0.08 (−0.08, 0.23)	**0.14 (0.03, 0.24)**
Normal ABI (0.9–1.3)	0.00 (−0.06, 0.06)	0.03 (−0.07, 0.13)	0.03 (−0.08, 0.13)

*Data are continuous NRI (95% confidence intervals). The bold values indicate statistical significance*.

*Intermediate risk: 10-year ASCVD risk 7.5% to 19.9% by the PCEs; High risk: 10-year ASCVD risk ≥ 20% by the PCEs*.

*Data cannot be shown due to a limited number of participants with high risk and baPWV ≤ 25th percentile (n = 45).

*NRI, net reclassification index; CVD, cardiovascular diseases; HDL, high-density lipoprotein; Lp (a), lipoprotein(a); apoB, apolipoprotein B; apoA-I, apolipoprotein A-I; UACR, urinary albumin-to-creatinine ratio; baPWV, brachial-ankle pulse wave velocity; CIMT, carotid intima-media thickness; ABI, ankle-brachial index; ECG, electrocardiogram; QTc interval, corrected QT interval; ASCVD, atherosclerotic cardiovascular disease; PCEs, Pooled Cohort Equations*.

As shown in Table 5, lipoprotein(a) ≤ 10th percentile and CIMT ≤ 25th percentile provided the greatest downward change in pre-test to post-test risk, both with a mean multivariable-adjusted DLR of 0.41 in participants with high predicted ASCVD risk. This equates to a 59% reduction in relative risk compared with that expected from traditional risk factors. Among participants at high risk, lipoprotein(a) ≤ 25th percentile (DLR 0.67) and normal ECG (DLR 0.75) also provided significant downward changes in pre-test to post-test risk. In participants at intermediate risk, lipoprotein(a) ≤ 10th (DLR 0.78) also showed a significant downregulation of CVD risk, followed by apoA-I ≥75th percentile (DLR 0.80) and CIMT ≤ 25th percentile (DLR 0.87).

**Table 5:** Multivariable adjusted DLR.

**Negative risk markers**	**Total**	**Intermediate risk**	**High risk**
**Non-traditional lipids**			
Lp(a) ≤ 25th percentile (9 mg/dL)	0.85 ± 0.01	0.91 ± 0.01	0.67 ± 0.03
Lp(a) ≤ 10th percentile (5 mg/dL)	0.68 ± 0.01	0.78 ± 0.01	0.41 ± 0.02
apoB ≤ 25th percentile (0.81 g/L)	0.99 ± 0.00	0.91 ± 0.03	0.81 ± 0.04
apoB ≤ 10th percentile (0.69 g/L)	1.12 ± 0.02	1.00 ± 0.00	0.88 ± 0.01
apoA-I ≥ 75th percentile (1.43 g/L)	0.95 ± 0.02	0.80 ± 0.06	1.17 ± 0.06
apoA-I ≥ 90th percentile (1.60 g/L)	1.00 ± 0.00	1.03 ± 0.00	1.12 ± 0.02
**Urinary ACR**			
<30 mg/g	0.96 ± 0.03	0.98 ± 0.02	0.91 ± 0.04
<10 mg/g	0.94 ± 0.03	0.93 ± 0.03	0.91 ± 0.03
**Electrocardiogram**			
Normal ECG	0.89 ± 0.01	0.88 ± 0.02	0.75 ± 0.03
Normal QTc (men: 390–450 ms, women: 390–460 ms)	0.92 ± 0.02	0.93 ± 0.02	0.86 ± 0.03
**Measurements of atherosclerosis**			
baPWV ≤ 25th percentile (1,350 cm/s)	1.34 ± 0.09	1.35 ± 0.07	/*
CIMT ≤ 25th percentile (0.5 mm)	0.80 ± 0.05	0.87 ± 0.01	0.41 ± 0.02
Normal ABI (0.9–1.3)	1.01 ± 0.00	1.02 ± 0.00	0.99 ± 0.00

*Data are means ± standard deviations*.

*Intermediate risk: 10-year ASCVD risk 7.5% to 19.9% by the PCEs; High risk: 10-year ASCVD risk ≥ 20% by the PCEs*.

*Data cannot be shown due to a limited number of participants with high risk and baPWV ≤ 25th percentile (n = 45).

*DLR, diagnostic likelihood ratios; CVD, cardiovascular diseases; HDL, high-density lipoprotein; Lp (a), lipoprotein(a); apoB, apolipoprotein B; apoA-I, apolipoprotein A-I; UACR, urinary albumin-to-creatinine ratio; baPWV, brachial-ankle pulse wave velocity; CIMT, carotid intima-media thickness; ABI, ankle-brachial index; ECG, electrocardiogram; QTc interval, corrected QT interval; ASCVD, atherosclerotic cardiovascular disease; PCEs, Pooled Cohort Equations*.

Finally, we did a sensitivity analysis using tertiles of PCE score in the study population to define intermediate (3.6 to 11.3%) and high (≥11.4%) risk groups. Results were largely unchanged (Supplementary Tables 1–3). In addition, we combined the 3 potential negative risk markers including lipoprotein(a) ≤ 5 mg/dL, normal ECG, and CIMT ≤ 0.5 mm and re-did the analysis using the number of negative risk markers (Supplementary Tables 4–7). When having ≥2 negative risk markers, the associations, post- vs. pre-test risks, and down-reclassifications of CVD risks were all improved significantly in both intermediate- and high-risk participants.

## Discussion

Using data from a large cohort of community adults aged ≥40 years followed up for a median of 4.5 years, we evaluated several non-traditional negative risk markers in associations with the development of incident CVD and in risk downgrade of CVD events. We found that lipoprotein(a) ≤ 10th percentile (5 mg/dL), normal ECG, normal QTc, and CIMT ≤ 25th percentile (0.5 mm) were significantly associated with a reduced CVD risk, had moderate CVD risk reclassification, and provided downward changes in pre- to post-test risk on top of the traditional CVD risk factors, especially in high-risk population. Therefore, CVD risk assessment should take into account both traditional and non-traditional risk factors and negative risk markers such as a low lipoprotein(a) level, normal ECG, and a low CIMT might be considered in adults categorized as high risk by traditional risk evaluation tools before making decisions of preventive treatment.

Lipoprotein(a) is a major oxidized phospholipids (OxPL) carrier in human circulation and mediates clearance of these proinflammatory factors. However, high levels of lipoprotein(a) with its OxPL load recirculating into the vasculature may turn into harm (16). Previous epidemiological studies have reported that elevated plasma lipoprotein(a) was an independent risk factor for cardiovascular disease (17, 18). In addition, studies have shown that lipoprotein(a) improved the reclassification of predicted CVD risk when added to the PCE score or traditional risk factors (18, 19). Therefore, the 2019 ACC/AHA guideline on the primary prevention of cardiovascular diseases defined a high level of lipoprotein(a) as one of the risk enhancers (20). Although previous studies have long focused on the association of high levels of lipoprotein(a) with increased cardiovascular risk, a recent study found that 1-SD genetically lowered lipoprotein(a) level was associated with a 29% lower risk of coronary heart disease and a 13% lower risk of stroke (21). However, a lipoprotein(a) <25th percentile (6 mg/dl) failed to downshift the predicted risk of CVD in an elderly US population (22). A similar finding was observed in the current study for a lipoprotein(a) ≤ 25th percentile (9 mg/dl), whereas the predicted risk of CVD could be moderately downshifted when lipoprotein(a) was at a lower level (i.e., ≤ 5 mg/dl at 10th percentile). Pronounced differences across ethnicities with regard to lipoprotein(a) levels have been well-demonstrated (23) and further studies are warranted in other ethnic populations.

Previous studies have reported that ECG abnormalities among older adults were associated with an increased risk of CVD outcomes (24, 25). The US Preventive Services Task Force (USPSTF) recently updated its recommendations on ECG and recommended against screening with resting or exercise ECG for the prevention of CVD in asymptomatic adults at low risk (26). Findings from the current study may have provided a new angle at this by demonstrating that in the high-risk population predicted by traditional risk factors, normal ECG could correctly reclassify some participants to a lower predicted CVD risk category. In addition, we previously reported that a prolonged QTc interval was associated with an increased risk of CVD (27). We went further in the current study to demonstrate that a normal QTc interval could correctly downshift the predicted CVD risk beyond traditional risk factors in Chinese adults, which has important implications in risk prediction to guide preventive treatment.

We found that CIMT ≤ 0.5 mm resulted in a significant change in pre-test to post-test risk for CVD. CIMT is the thickness of the intimal and medial layer of the carotid artery wall. It can be measured non-invasively using ultrasound imaging and is considered a marker for the early stage of atherosclerosis. High CIMT independently predicts cardiovascular events (28). However, the reclassification of CVD risk by CIMT is inconsistent between studies. One study revealed that adding CIMT to traditional risk assessment tool did not lead to a clinically important improvement in risk prediction of myocardial infarction or stroke (29). However, another study demonstrated that adding CIMT to traditional risk factors significantly improved risk classification in older women, but not in older men (30). In addition, it was reported that CIMT modestly improved risk prediction for CVD in older adults mainly by down-classifying risk in those without CVD (31). Findings from the Multi-Ethnic Study of Atherosclerosis (MESA) revealed that low CIMT showed the best performance in reducing predicted CVD risk only after CAC = 0 (7). Findings from the current study also demonstrated better risk reduction by low CIMT compared with other potential negative risk markers.

The strengths of the current study included the large sample of community adults with well-characterized CVD risk and the comprehensive evaluation of multiple potential negative risk markers being compared directly for risk prediction in a single cohort. It also has several limitations. First, a limited number of CVD events were recorded due to a relatively short duration of follow-up. Second, all the tests and measurements were conducted only once. Although CIMT was measured by an experienced ultrasound examiner, two examinations for each participant should have been conducted by two independent examiners. The CIMT was measured only at the common carotid arteries. However, a comprehensive evaluation of CIMT at the common carotid arteries, the carotid bifurcation, and the internal carotid artery is the best way to display atherosclerosis burden (32). Third, the PCEs used in the current study to predict ASCVD risk by traditional risk factors were developed in western populations. The prediction for ASCVD risk in China (China-PAR) equations should have been used to estimate the CVD risk in Chinese population (9). However, we did not have information on family history of CVD which is required in the China-PAR equations. Fourth, other potential negative risk markers such as CAC = 0 were not evaluated in the current study due to lack of information. Finally, the generalizability of the findings is limited to Chinese community residents aged ≥40 years.

In conclusion, lipoprotein(a) ≤ 5 mg/dL, normal ECG, and CIMT ≤ 0.5 mm moderately downshifted predicted ASCVD risk in Chinese adults with high risk predicted based on traditional risk factors. The role of negative non-traditional risk markers in CVD risk prediction warrants further investigation.

## Data Availability Statement

The datasets presented in this article are not readily available because the IRB has requested that currently, the dataset should be used by the research team members only. If the dataset has to be accessed to verify the results, the request can be directed to the corresponding author. Requests to access the datasets should be directed to YX, jane.yuxu@gmail.com.

## Ethics Statement

The studies involving human participants were reviewed and approved by Institutional Review Board of the Ruijin Hospital affiliated to Shanghai Jiaotong University School of Medicine. The patients/participants provided their written informed consent to participate in this study.

## Author Contributions

LB, JN, and SWu: conceptualization, formal analysis, and writing—original draft. RZ, MX, JL, TW, ZZ, SWa, HL, MD, DZ, and YC: acquisition of data and revision of the manuscript for intellectual content. WW and GN: supervision, funding acquisition, and revision of the manuscript for intellectual content. YB, ML, and YX: conceptualization, formal analysis, funding acquisition, and critical revision of the manuscript for important intellectual content. All authors contributed to the article and approved the submitted version.

## Funding

This work was supported by the grants from the National Natural Science Foundation of China (81870560, 81970691, 81941017, 81770842, 81970706, 82022011, and 82070880), the Shanghai Municipal Government (18411951800), the Shanghai Shenkang Hospital Development Center (SHDC12019101, SHDC2020CR1001A, and SHDC2020CR3069B), the Shanghai Jiaotong University School of Medicine (DLY201801), and the Ruijin Hospital (2018CR002).

## Conflict of Interest

The authors declare that the research was conducted in the absence of any commercial or financial relationships that could be construed as a potential conflict of interest.

## Publisher's Note

All claims expressed in this article are solely those of the authors and do not necessarily represent those of their affiliated organizations, or those of the publisher, the editors and the reviewers. Any product that may be evaluated in this article, or claim that may be made by its manufacturer, is not guaranteed or endorsed by the publisher.
