# Supplementary material for: Negative Risk Markers for Cardiovascular Risk Evaluation in Chinese Adults
Source: Front Cardiovasc Med. 2022 Mar 15;9:800671. doi: 10.3389/fcvm.2022.800671 (PMC8964789; doi:10.3389/fcvm.2022.800671)
Supplement: Supplementary file 1 [file Data_Sheet_1.docx]

**SUPPLEMENTAL MATERIALS**

Supplemental Table 1. The associations of negative risk markers with CVD risk using alternative cutoffs for intermediate risk and high risk

Supplemental Table 2. Continuous NRI for CVD risk by adding each negative risk markers to a basic model with traditional CVD risk factors using alternative cutoffs for intermediate risk and high risk

Supplemental Table 3. Multivariable-adjusted DLR using alternative cutoffs for intermediate risk and high risk

Supplemental Table 4. The development of CVD events in participants with different numbers of negative risk markers

Supplemental Table 5. The associations of negative risk marker combinations with CVD risk

Supplemental Table 6. Continuous NRI for CVD risk by adding negative risk marker combinations to a basic model with traditional CVD risk factors

Supplemental Table 7. Multivariable adjusted DLR for negative risk marker combinations

Supplemental Table 1. The associations of negative risk markers with CVD risk using alternative cutoffs for intermediate risk and high risk

| Negative risk markers | Intermediate risk (n=2520) | | High risk (n=2580) | |
| --- | --- | --- | --- | --- |
|  | Model 1 | Model 2 | Model 1 | Model 2 |
| **Non-traditional lipids** | | | | |
| Lp(a) ≤ 25th percentile (9 mg/dL) | 0.97 (0.62, 1.51) | 0.97 (0.62, 1.52) | 0.76 (0.56, 1.02) | **0.72 (0.53, 0.98)** |
| Lp(a) ≤ 10th percentile (5 mg/dL) | 0.92 (0.49, 1.72) | 0.93 (0.49, 1.74) | 0.60 (0.37, 0.97) | **0.56 (0.35, 0.91)** |
| apoB ≤ 25th percentile (0.81 g/L) | 0.67 (0.39, 1.17) | 0.54 (0.29, 1.02) | 0.86 (0.62, 1.18) | 0.92 (0.64, 1.34) |
| apoB ≤ 10th percentile (0.69 g/L) | 0.87 (0.42, 1.81) | 0.76 (0.34, 1.69) | 0.85 (0.53, 1.37) | 0.93 (0.56, 1.55) |
| apoA-I ≥ 75th percentile (1.43 g/L) | 1.04 (0.66, 1.62) | 0.88 (0.52, 1.50) | 1.03 (0.77, 1.37) | 1.00 (0.70, 1.42) |
| apoA-I ≥ 90th percentile (1.60 g/L) | 1.32 (0.74, 2.32) | 1.20 (0.62, 2.34) | 1.01 (0.66, 1.53) | 0.95 (0.58, 1.55) |
| **Urinary ACR** | | | | |
| < 30 mg/g | 1.28 (0.56, 2.93) | 1.29 (0.56, 2.98) | **0.47 (0.34, 0.66)** | **0.54 (0.38, 0.76)** |
| < 10 mg/g | 0.73 (0.48, 1.13) | 0.74 (0.48, 1.14) | **0.70 (0.53, 0.91)** | 0.78 (0.59, 1.03) |
| **Electrocardiogram** | | | | |
| Normal ECG | 0.97 (0.65, 1.46) | 0.98 (0.65, 1.47) | **0.67 (0.50, 0.90)** | **0.72 (0.54, 0.96)** |
| Normal QTc (men: 390-450 ms, women: 390-460ms) | **0.65 (0.42, 0.99)** | **0.64 (0.42, 0.99)** | **0.61 (0.47, 0.79)** | **0.65 (0.50, 0.84)** |
| **Measurements of atherosclerosis** |  |  |  |  |
| CIMT ≤ 25th percentile (0.5 mm) | 0.88 (0.58, 1.34) | 0.88 (0.58, 1.33) | **0.53 (0.33, 0.83)** | **0.53 (0.34, 0.84)** |
| baPWV ≤ 25th percentile (1350 cm/s) | 1.38 (0.83, 2.27) | 1.43 (0.84, 2.44) | 0.91 (0.52, 1.58) | 1.22 (0.69, 2.17) |
| Normal ABI (0.9-1.3) | 1.40 (0.62, 3.20) | 1.41 (0.62, 3.23) | 0.96 (0.65, 1.44) | 1.00 (0.67, 1.49) |

Data are hazard ratios (95% confidence intervals). The bold values indicate statistical significance.

Intermediate risk: 10-Year ASCVD (atherosclerotic cardiovascular disease) risk 3.6% to 11.3% by the PCEs; High risk: 10-year ASCVD risk ≥ 11.4% by the PCEs.

Model 1: adjusted for age, sex.

Model 2: further adjusted for smoking status, diabetes, total cholesterol, HDL-c, systolic blood pressure, antihypertensive drugs.Abbreviations: CVD, cardiovascular diseases; Lp(a), lipoprotein(a); apoB, apolipoprotein B; apoA-I, apolipoprotein A-I; UACR, urinary albumin-to-creatinine ratio; ECG, electrocardiogram; QTc interval, corrected QT interval; CIMT, carotid intima-media thickness; baPWV, brachial-ankle pulse wave velocity; ABI, ankle-brachial index; HDL-c, high-density lipoprotein cholesterol; PCEs, pooled cohort equations.

Supplemental Table 2. Continuous NRI for CVD risk by adding each negative risk markers to a basic model with traditional CVD risk factors using alternative cutoffs for intermediate risk and high risk

| Negative Risk Markers | Intermediate risk (n=2520) | High risk (n=2580) |
| --- | --- | --- |
| **Non-traditional lipids** | | |
| Lp(a) ≤ 25th percentile (9 mg/dL) | 0.04 ( -0.13, 0.22) | **0.15 (0.04, 0.26)** |
| Lp(a) ≤ 10th percentile (5 mg/dL) | 0.05 ( -0.07, 0.17) | **0.13 (0.05, 0.20)** |
| apoB ≤ 25th percentile (0.81 g/L) | 0.18 (0.00, 0.36) | 0.01 ( -0.11, 0.14) |
| apoB ≤ 10th percentile (0.69 g/L) | 0.04 ( -0.13, 0.22) | -0.01 ( -0.13, 0.11) |
| apoA-I ≥ 75th percentile (1.43 g/L) | 0.09 ( -0.10, 0.28) | 0.04 ( -0.09, 0.17) |
| apoA-I ≥ 90th percentile (1.60 g/L) | -0.09 ( -0.28, 0.10) | 0.08 ( -0.05, 0.21) |
| **Urinary ACR** | | |
| < 30 mg/g | 0.03 ( -0.10, 0.16) | **0.15 (0.05, 0.25)** |
| < 10 mg/g | 0.16 ( -0.02, 0.34) | **0.21 (0.09, 0.34)** |
| **Electrocardiogram** | | |
| Normal electrocardiogram | 0.06 ( -0.13, 0.25) | **0.22 (0.10, 0.34)** |
| Normal QTc (men: 390-450 ms, women: 390-460ms) | 0.17 ( -0.01, 0.34) | **0.24 (0.12, 0.37)** |
| **Measurements of atherosclerosis** | | |
| CIMT ≤ 25th percentile (0.5 mm) | 0.08 ( -0.10, 0.27) | **0.16 (0.03, 0.22)** |
| baPWV ≤ 25th percentile (1350 cm/s) | 0.09 ( -0.10, 0.28) | **0.16 (0.04, 0.28)** |
| Normal ABI (0.9-1.3) | 0.04 ( -0.05, 0.14) | 0.01 ( -0.07, 0.10) |

Values are NRI (95% CI). The bold values indicate statistical significance.

Intermediate risk: 10-Year ASCVD Risk 3.6% to 11.3% by the PCEs; High risk: 10-year ASCVD risk ≥ 11.4% by the PCEs.

NRI: net reclassification index; CI: confidence interval; other abbreviations as in Supplemental Table 1.

Supplemental Table 3. Multivariable adjusted DLR using alternative cutoffs for intermediate risk and high risk

| Negative risk markers | Intermediate risk(n=2520) | High risk (n=2580) |
| --- | --- | --- |
| **Non-traditional lipids** |  |  |
| Lp(a) ≤ 25th percentile (9 mg/dL) | 0.97 ± 0.01 | 0.76 ± 0.02 |
| Lp(a) ≤ 10th percentile (5 mg/dL) | 0.93 ± 0.00 | 0.57 ± 0.02 |
| apoB ≤ 25th percentile (0.81 g/L) | 0.71 ± 0.07 | 0.78 ± 0.05 |
| apoB ≤ 10th percentile (0.69 g/L) | 0.82 ± 0.02 | 0.91 ± 0.01 |
| apoA-I ≥ 75th percentile (1.43 g/L) | 0.89 ± 0.03 | 1.00 ± 0.00 |
| apoA-I ≥ 90th percentile (1.60 g/L) | 1.17 ± 0.03 | 0.93 ± 0.01 |
| **Urinary ACR** |  |  |
| < 30 mg/g | 1.01 ± 0.01 | 0.93 ± 0.04 |
| < 10 mg/g | 0.93 ± 0.03 | 0.93 ± 0.03 |
| **Electrocardiogram** |  |  |
| Normal electrocardiogram | 0.97 ± 0.00 | 0.78 ± 0.02 |
| Normal QTc (men: 390-450 ms, women: 390-460ms) | 0.90 ± 0.02 | 0.88 ± 0.02 |
| **Measurements of atherosclerosis** |  |  |
| CIMT ≤ 25th percentile (0.5 mm) | 0.91 ± 0.01 | 0.57 ± 0.02 |
| baPWV ≤ 25th percentile (1350 cm/s) | 1.33 ± 0.09 | 1.20 ± 0.02 |
| Normal ABI (0.9-1.3) | 1.02 ± 0.00 | 1.00 ± 0.00 |

Data are means ± standard deviations.

Intermediate risk: 10-Year ASCVD Risk 3.6% to 11.3% by the PCEs; High risk: 10-year ASCVD risk ≥ 11.4% by the PCEs.

DLR: diagnostic likelihood ratios; other abbreviations as in Supplemental Table 1.

Supplemental Table 4. The development of CVD events in participants with different numbers of negative risk markers

| Number of negative risk markers | Number of participants, n (%) | Incident CVD events, n (%) |
| --- | --- | --- |
| 0 | 2440 (32.16) | 204 (8.36) |
| 1 | 3487 (45.97) | 164 (4.12) |
| 2 | 1487 (19.60) | 46 (2.89) |
| 3 | 172 (2.27) | 2 (1.16) |

Negative risk markers included lipoprotein(a) ≤5 mg/dL, normal ECG, and CIMT ≤0.5 mm.

Abbreviations as in Supplemental Table 1.

Supplemental Table 5. The associations of negative risk marker combinations with CVD risk

| Number of negative risk markers | Total | | Intermediate risk | | High risk | |
| --- | --- | --- | --- | --- | --- | --- |
|  | Model 1 | Model 2 | Model 1 | Model 2 | Model 1 | Model 2 |
| 0 (n=2440) | 1.00 | 1.00 | 1.00 | 1.00 | 1.00 | 1.00 |
| 1 (n=3487) | **0.70 (0.57, 0.87)** | **0.73 (0.59, 0.91)** | 1.07 (0.73, 1.55) | 1.09 (0.75, 1.59) | **0.50 (0.35, 0.70)** | **0.52 (0.36, 0.73)** |
| ≥2 (n=1659) | **0.56 (0.40, 0.78)** | **0.60 (0.43, 0.84)** | **0.41 (0.19, 0.92)** | **0.40 (0.18, 0.90)** | **0.38 (0.17, 0.82)** | **0.39 (0.18, 0.84)** |

Data are hazard ratios (95% confidence intervals). The bold values indicate statistical significance.

Negative risk markers included lipoprotein(a) ≤5 mg/dL, normal ECG, and CIMT ≤0.5 mm.

Intermediate risk: 10-year ASCVD risk 7.5% to 19.9% by the PCEs; High risk: 10-year ASCVD risk ≥ 20% by the PCEs.

Model 1: adjusted for age and sex.

Model 2: further adjusted for smoking status, diabetes, total cholesterol, HDL-c, systolic blood pressure, antihypertensive drugs.

Abbreviations as in Supplemental Table 1.

Supplemental Table 6. Continuous NRI for CVD risk by adding negative risk marker combinations to a basic model with traditional CVD risk factors

| Number of negative risk markers | Total | Intermediate risk | High risk |
| --- | --- | --- | --- |
| 1 (n=3487) | **0.30 (0.20, 0.41)** | 0.01 (-0.18, 0.20) | **0.38 (0.22, 0.53)** |
| ≥2 (n=1659) | **0.22 (0.10, 0.34)** | **0.24 (0.05, 0.44)** | **0.20 (0.07, 0.32)** |

Values are NRI (95% confidence interval). The bold values indicate statistical significance.

Negative risk markers included lipoprotein(a) ≤5 mg/dL, normal ECG, and CIMT ≤0.5 mm.

Intermediate risk: 10-year ASCVD risk 7.5% to 19.9% by the PCEs; High risk: 10-year ASCVD risk ≥ 20% by the PCEs.

NRI: net reclassification index; other abbreviations as in Supplemental Table 1.

Supplemental Table 7. Multivariable adjusted DLR for negative risk marker combinations

| Number of negative risk markers | Total | Intermediate risk | High risk |
| --- | --- | --- | --- |
| 1 (n=3487) | 0.87 ± 0.03 | 1.05 ± 0.01 | 0.64 ± 0.03 |
| ≥2 (n=1659) | 0.70 ± 0.07 | 0.46 ± 0.05 | 0.38 ± 0.03 |

Data are means ± standard deviations.

Negative risk markers included lipoprotein(a) ≤5 mg/dL, normal ECG, and CIMT ≤0.5 mm.

Intermediate risk: 10-year ASCVD risk 7.5% to 19.9% by the PCEs; High risk: 10-year ASCVD risk ≥ 20% by the PCEs.

DLR: diagnostic likelihood ratios; other abbreviations as in Supplemental Table 1.
